# Supplementary material for: Patient perceptions by race of educational animations about living kidney donation made for a diverse population
Source: PLoS One. 2022 Sep 15;17(9):e0266867. doi: 10.1371/journal.pone.0266867 (PMC9477306; doi:10.1371/journal.pone.0266867)
Supplement: Attachment — Submitted filename: Response to Reviewers.docx [file pone.0266867.s001.docx]

July 25, 2022

Re: Resubmission of manuscript Patient Perceptions by Race of Educational Animations About Living Kidney Donation Made for a Diverse Population, PONE-D-22-09153

The Editor:

Dr. Michael Scott Brewer

PLOS ONE

East Carolina University

100 E 5^th^ St., Howell Science Complex N407, Greenville, NC 27858

Dear Dr. Brewer,

Thank you for the opportunity to revise our manuscript named, *Patient Perceptions by Race of Educational Animations About Living Kidney Donation Made for a Diverse Population*. We

appreciate the careful review and constructive suggestions. We believe that the manuscript is

substantially improved after making the suggested edits.

Following this letter are the reviewer comments with our responses in italics,

including how and where the text was modified. Changes made in the manuscript are marked

using track changes. The revision has been developed in consultation with all coauthors, and each author has given approval to the final form of this revision.

Last, our revised data availability statement, that addresses our ethical limitation in sharing sensitive data, is included in our Statements & Declarations section.

Thank you for your consideration.

Sincerely,

Maria M. Keller, MS

**REVIEWER 2**

**Title**

*No revisions requested.*

**Abstract**

*No revisions requested.*

**Introduction**

I note the authors state that “Black Americans are disproportionately burdened by kidney failure, but are far less likely than any other racial or ethnic groups to undergo LDKT”

Are they classifying Black Americans as a separate race or ethnicity? If so, what do they mean by these terms? I note race is used hereafter.

*Response: Thank you for noting the need for clarification. We have indicated in the introduction that our term Black American encompasses individuals who self-identify as Black or as African American. This definition was reinforced in the methods section.*

I note that the authors state “Black patients may need to reach out to a larger pool of potential living donors, since Black individuals who come forward as potential living donors are more likely to have disqualifying medical conditions”. Could the authors explain why Black people are only limited to kidneys from Black people?

*Response: We did not mean to imply that Black people are limited to kidneys only from Black people and have revised the statement to better reflect the original intent that expanding the donor pool overall is needed.*

I note that the transcripts being reviewed were already available when creating the first version of the videos. Could the authors explain why the original video creation may have excluded the views of Black participants, and why this re-review was needed?

*Response: We have revised the introduction to more clearly indicate that we originally created a single educational product that was intended to affect most audiences; however, different message strategies and channel choices may be required to optimally influence different sub-groups. The decision on how to adapt should be made in the context of the degree of actual heterogeneity with regard to influences on behavior, channel choices, and responsiveness to different message executions.*

**Methods**

Do the authors believe their gender, race, or ethnicity affected the data collected? If so, how? Some sentences of reflection would be appreciated

*Response: Since all of the interviewers were female and all but one were non-Hispanic White, and the sole interviewer that was Black conducted a paucity of interviews, we were unable to investigate differences in data collection by interviewer demographic. We have indicated this limitation in the paper.*

**Results**

I note that Black patients had other demographic factors that were significantly different from non-Black patients. Could these other demographic factors be confounding results? Could the differences seen be a derivative of poverty and not race?

*Response: Thank you for illuminating this important point. We have updated our limitations section to indicate the following: Differences that were found between race groups could also be a function of varying demographic factors that were different between Black and non-Black individuals, such as employment and income status, rather than race.*

**Discussion**

I note the authors make presumptions about the cognitive barriers present among Black patients – is there is a reason that the authors didn’t explore the rationale during the interview?

*Response: We updated our methods to indicate that the interviews were semi-structured and that our analysis included probes related to the relevant questions. We have also added to the limitations section the possibility that we did not delve deeply into rationale around thoughts, feelings, and beliefs about a particular topic, possibly resulting in incomplete understanding.*

**Data Availability Statement is required.**

*Response: Our data availability statement is now provided in the manuscript. Limited data can be made available to researchers who meet the criteria for access to confidential data. Due to the qualitative nature of these data, the interview transcripts contain personal information that potentially identifies participants and would breach participant confidentiality if made publicly available. Data requests may be sent to the corresponding author. We will make our data available to researchers requesting the data directly from us. In that case, we will facilitate the submission of a short research proposal to the University at Buffalo, which also approved the original collection of the data. Once approved, we will happily share these data with future researchers for re-analysis. The University at Buffalo Internal Review Board can be reached at (716) 888-4888.*

*By way of context, this study interviewed kidney failure patients and social network members at a single hospital. Release our data publicly is problematic since logical supposition of who the source is by members of the same community is possible and could jeopardize the anonymity of our participants and social network ties that arises from their shared history with others relating to kidney donation requests. In selecting particular quotes for the paper (grouped by theme rather than participant), we have ensured that no potentially identifiable information is included, but this information is woven throughout the interview transcripts and any suitably redacted or anonymized transcripts would be so unserviceably thin that they would be devoid of meaningful content.*
